# Genetic Manipulation of the Ergot Alkaloid Pathway in *Epichloë festucae* var. *lolii* and Its Effect on Black Beetle Feeding Deterrence

**DOI:** 10.3390/toxins13020076

**Published:** 2021-01-20

**Authors:** Debbie Hudson, Wade Mace, Alison Popay, Joanne Jensen, Catherine McKenzie, Catherine Cameron, Richard Johnson

**Affiliations:** 1 AgResearch Limited, Grasslands Research Centre, Private Bag 11008, Palmerston North, New Zealand; debbie.hudson@agresearch.co.nz (D.H.); wade.mace@agresearch.co.nz (W.M.); Catherine.McKenzie@plantandfood.co.nz (C.M.); 2 AgResearch Limited, Ruakura Research Centre, Private Bag 3123, Hamilton, New Zealand; alison.popay@agresearch.co.nz (A.P.); joanne.jensen@agresearch.co.nz (J.J.); catherine.cameron2@agresearch.co.nz (C.C.)

**Keywords:** *Epichloë*, ergot alkaloid, ergovaline, black beetle, *Heteronychus arator*, perennial ryegrass, symbiosis, endophyte, secondary metabolite

## Abstract

*Epichloë* endophytes are filamentous fungi (family Clavicipitaceae) that live in symbiotic associations with grasses in the sub family Poöideae. In New Zealand, *E. festucae* var. *lolii* confers significant resistance to perennial ryegrass (*Lolium perenne*) against insect and animal herbivory and is an essential component of pastoral agriculture, where ryegrass is a major forage species. The fungus produces in planta a range of bioactive secondary metabolites, including ergovaline, which has demonstrated bioactivity against the important pasture pest black beetle, but can also cause mammalian toxicosis. We genetically modified *E. festucae* var. *lolii* strain AR5 to eliminate key enzymatic steps in the ergovaline pathway to determine if intermediate ergot alkaloid compounds can still provide insecticidal benefits in the absence of the toxic end product ergovaline. Four genes (*dmaW*, *easG*, *cloA*, and *lpsB*) spanning the pathway were deleted and each deletion mutant was inoculated into five different plant genotypes of perennial ryegrass, which were later harvested for a full chemical analysis of the ergot alkaloid compounds produced. These associations were also used in a black beetle feeding deterrence study. Deterrence was seen with just chanoclavine present, but was cumulative as more intermediate compounds in the pathway were made available. Ergovaline was not detected in any of the deletion associations, indicating that bioactivity towards black beetle can be obtained in the absence of this mammalian toxin.

## 1. Introduction

*Epichloë* endophytes are filamentous fungi, belonging to the family *Clavicipitaceae,* which live in symbiotic associations with grasses in the sub family Poöideae [1,2]. In New Zealand, *E. festucae* var. *lolii* confers significant resistance to perennial ryegrass (*Lolium perenne*) against insect and animal herbivory and, as such, is an essential component of pastoral agriculture [3,4,5], where ryegrass is a major forage species. African black beetle (*Heteronychus arator*) is an introduced pest in northern New Zealand and is responsible for devasting losses to the pasture industry, costing farmers up to $242 M annually [6]. A number of *E. festucae* var. *lolii* strains provide biological control against this serious pest [7,8] through the production of secondary metabolites in planta [9]. Ergovaline, which is the best characterised *Epichloë*-produced ergot alkaloid [9,10,11,12,13], has demonstrated insecticidal activity against black beetle [14,15], but can also cause mammalian toxicosis [10,12,16,17].

**Figure 1:**
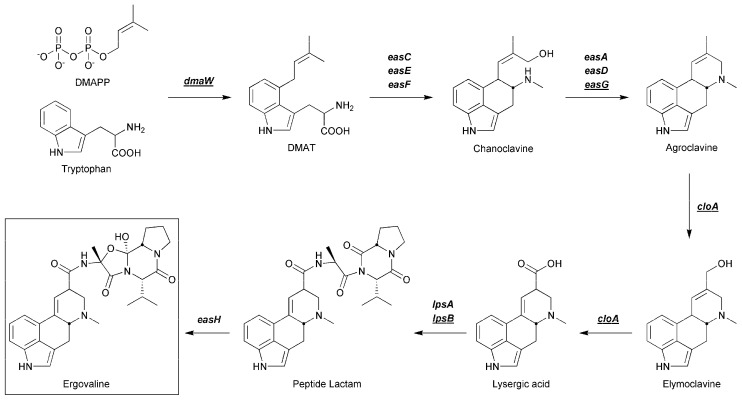
Simplified ergovaline pathway showing pathway intermediates and the key genes proposed for each biosynthetic step. DMAPP = dimethylallyl pyrophosphate, DMAT = dimethylallyl tryptophan. Underlined genes were deleted in this study. Ergovaline (boxed) is a mammalian toxin with bioactivity towards black beetle. A comprehensive review of this pathway is reported in Young et al., 2015 [24].

The biosynthetic pathway responsible for ergot alkaloid production has been well characterized from a number of fungi [11,13,18,19,20,21], including the ergovaline pathway from *Epichloë* [9,22]. While the end product of this pathway is ergovaline, intermediate ergot alkaloid compounds are also produced (Figure 1) and the genes responsible for each enzymatic step in the ergovaline pathway have been identified (Figure 1) [9,23,24].

Functional analysis, through genetic modification, of key genes in this pathway has been achieved for several fungi, including *Epichloë*, and has provided a detailed understanding of the key biosynthetic steps required for ergot alkaloid biosynthesis [20,24]. The gene *dmaW* encodes dimethylallyl tryptophan synthase, the first enzyme in the ergot alkaloid pathway [25], and as such, deletion of *dmaW* will lead to the elimination of all ergot alkaloids. The gene *easG* encodes an NADPH-dependent reductase involved in converting, via a non-enzymatic adduct with reduced glutathione, the simple ergot alkaloid chanoclavine to agroclavine [26,27]. Deletion of this gene should thus lead to the accumulation of chanoclavine in the absence of other ergot alkaloid compounds. The gene *cloA* encodes a cytochrome P450 monooxygenase, which is proposed to be responsible for the oxidation of both agroclavine (to form elymoclavine) and elymoclavine (to form lysergic acid) [28]. Deletion of this gene is thus predicted to lead to the accumulation of chanoclavine and agroclavine in the absence of more complex ergot alkaloids. The genes *lpsA* and *lpsB* encode the large and small subunits of a large non-ribosomal peptide synthetase, which is responsible for converting lysergic acid to ergovaline, the last step in the pathway [22,29]. Deletion of either of these genes leads to the accumulation of the clavines group and lysergic acid [22,29].

Despite ergovaline being strongly linked to bioactivity against black beetle [14,15], studies with perennial ryegrass infected with a Δ*lpsB E. festucae* mutant surprisingly showed that ergovaline was not required for feeding deterrence [30], suggesting that intermediate ergot alkaloid compounds, or a completely different alkaloid, are responsible. While ergovaline is a known mammalian toxin, the toxicity of the intermediates, and whether they also have bioactivity towards black beetle, is less well characterised. In this paper, we describe the outcome of gene deletions in the four key biosynthetic steps (described above) across the ergot alkaloid pathway in *E. festucae* var. *lolii* strain AR5. This strain is a commercially utilised (Endo5) *Epichloë* endophyte of perennial ryegrass [31] that usually produces low to moderate levels of ergovaline, but under adverse conditions, can produce levels that can cause mammalian toxicosis. We used these deletion mutants to engineer *Epichloë*-perennial ryegrass associations expressing a range of different ergot alkaloid compounds in a common host background. These associations were used in a black beetle bioactivity assay to determine whether ergot alkaloid intermediate compounds, such as the clavines or lysergic acid, can contribute to black beetle feeding deterrence in the absence of the mammalian toxin ergovaline.

## 2. Results and Discussion

### 2.1. Molecular Screening of Transformants to Identify Gene Deletions in the Ergot Alkaloid Pathway

*E. festucae* var. *lolii* strain AR5 wild type and hygromycin resistant transformants were nuclear purified by sub-culturing three times prior to genomic DNA extraction and screening by polymerase chain reaction (PCR) for each of the ergovaline gene deletion events illustrated in supplementary Figure S1. Transformants showing PCR products of the predicted size (Supplementary Table S1) for a gene deletion event or an ectopic integration, for each of the four ergot alkaloid pathway genes, were inoculated into perennial ryegrass for further analysis.

### 2.2. Analysis of Ergot Alkaloids in Gene Deletion Strains

Perennial ryegrass plants infected with AR5 wild type, two independent transformants corresponding to each of the ergot alkaloid pathway mutants (dmaW KO15 and KO20, easG KO3 and KO20, cloA KO6 and KO32, and lpsB KO10 and KO11) or ectopic integration controls (dmaW E1, easG E14, cloA E4, and lpsB E9), as well as uninfected plants, were analysed by mass spectrometry for the major ergot alkaloid compounds, in addition to peramine, an unrelated AR5 produced alkaloid that acted as a control for alkaloid production. No endophyte alkaloids were detected in uninfected plants, as expected, and peramine was detected in all endophyte-infected associations, indicating that genetic manipulation of the ergot alkaloid pathway did not otherwise impact secondary metabolism. In addition, all ectopic integrations had chemistry similar to the wild type strain, indicating that the transformation procedure had no impact on secondary metabolism production (Table 1; supplementary Table S2). For each of the AR5 ergot alkaloid pathway genes (Supplementary Table S3) deletions led to differences in the ergot alkaloid profiles detected (Table 1; supplementary Table S2). Deletion of the *dmaW* gene led to the absence of all ergot alkaloids (Table 1), which was expected given that this gene encodes the first committed step in the ergot alkaloid biosythetic pathway (Figure 1).

Deletion of *easG* led to the specific accumulation of chanoclavine (at levels similar to that seen in the wild type) in the absence of other ergot alkaloid compounds (Table 1) and fits the proposed biosythetic scheme, in which EasG is involved in the conversion of chanoclavine to agroclavine (Figure 1). The deletion of *cloA* led to the accumulation of chanoclavine (at elevated levels relative to the wild type) and agroclavine (at significantly higher levels than to the wild type) in the absence of elymoclavine, which was also not detected in wild type associations (Table 1). The absence of elymoclavine in the wild type associations suggests that this intermediate in the pathway is rapidly converted to lysergic acid, making detection difficult. However, for the *cloA* mutant, the absence of elymoclavine and lysergic acid confirms that CloA is likely to perform oxidation of both agroclavine and elymoclavine with the pathway terminating before lysergic acid (Figure 1), as previously proposed [24]. The deletion of *lpsB* led to a significant accumulation of lysergic acid (Table 1), which is not generally seen in wild type associations. This is similar to results obtained by others who performed earlier gene deletion studies on *lpsA* [29] and *lpsB* [22], both of which showed significant elevation of lysergic acid. All other earlier ergot alkaloids were detected at levels equivalent to the wild type.

### 2.3. Black Beetle Feeding Deterrence

**Figure 2:**
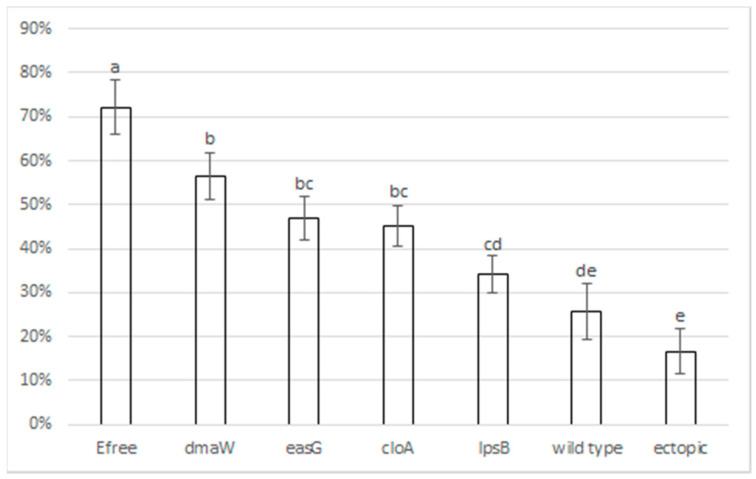
Binary logistic regression analysis of percentage black beetle feeding damage (*y*-axis) based upon combined scores of 1, 2, and 3 on all tillers for each association. Error bars represent two times the standard error of the estimate from the analysis. Different letters indicate a statistically significant difference (*p* < 0.05, least significant difference). Efree = uninfected perennial ryegrass.

The same perennial ryegrass associations described under Section 2.1 for mass spectrometry analysis were also utilised in a black beetle feeding experiment to determine if the altered secondary metabolite profiles of the ergot alkaloid gene deletion mutants had an impact on black beetle feeding. Damaged tillers were scored on a scale of 1–3 (Section 3.8) and the percentage of the total tillers with damage scores of 1, 2, and 3 (low, moderate, or severe damage, respectively) were compared between treatments. Black beetle feeding damage was significantly reduced in plants infected with wild type, all ergot alkaloid pathway deletion mutants, and the ectopic integration strains compared with uninfected perennial ryegrass (*p* < 0.05) (Figure 2). Interestingly, compared with uninfected associations, perennial ryegrass infected with the *dmaW* deletion mutant showed a significant (*p* < 0.05) reduction in black beetle feeding (Figure 2). As this mutant produces no ergot alkaloids, it suggests that additional unrelated endophyte secondary metabolites have some bioactivity against this pest. *E. festucae* var. *lolii* strain AR5 is characterised as producing peramine and early indole diterpenes in addition to ergovaline. However, peramine has been reported to not affect black beetle [14], so this bioactivity is most likely derived from the indole diterpene pathway, or an as yet uncharacterised compound, although it cannot be ruled out that *Epichloë* infection enhances the host plants’ immunity against black beetle by promoting endogenous defense responses mediated by the jasmonic acid (JA) pathway [32].

For the *easG* deletion treatments, overall, there was significantly less feeding damage (*p* < 0.05) compared with uninfected perennial ryegrass. However, this level of deterrence, although contributing to an upward trend, was not statistically different to the *dmaW* mutant, which suggests that chanoclavine, the early ergot alkaloid that solely accumulates in this association (Table 1), has only limited bioactivity against black beetle. This result is corroborated by feeding experiments using purified chanoclavine, which showed no bioactivity against black beetle at the tested concentrations (2.5, 5, 10, 50, and 100 µg/g) (unpublished results).

For the *cloA* deletion treatments, which accumulate agroclavine in addition to chanoclavine, feeding deterrence was similar to the *easG* treatments (Figure 2), which suggests that agroclavine, which accumulated significantly in the *cloA* mutant associations (Table 1), has little further activity in terms of black beetle feeding deterrence. Despite this, the clavine class of compounds, including agroclavine, have been shown to deter the feeding of fall armyworm (*Spodoptera frugiperda*) [33] at relatively higher concentrations than those determined here. It is therefore possible that, under certain conditions, such as those in the far north of New Zealand where drought is prevalent, clavines accumulate to higher levels and contribute to some black beetle feeding deterrence in the field.

The *lpsB* deletion treatments gave the highest levels of black beetle feeding deterrence compared with the wild type (Figure 2), and lysergic acid accumulated to levels not typically seen in wild type associations, suggesting that this compound may contribute significant bioactivity against black beetle.

Wild type associations and ectopic controls showed the greatest deterrence and, taken as a whole, the results from this study imply that bioactivity is cumulative across the entire ergot alkaloid pathway, up to and including ergovaline.

In summary, this research has clearly demonstrated that intermediate compounds in the ergot alkaloid pathway can provide bioactivity against the important pasture pest, black beetle, in the absence of the animal-toxic ergopeptine end product ergovaline. While these intermediate compounds may be less effective in reducing insect damage than ergovaline, this reduction may still offer significant protection to pastures, in the absence of animal toxicity, compared with endophyte free pastures [7]. However, while chanoclavine has been demonstrated to be non-toxic in a mouse assay [34], little information exists about the toxicity of the other clavine species, although agroclavine has been suggested to be cytotoxic at high concentrations [35]. Further studies on the potential toxicity of the clavines and lysergic acid are thus an important step to consider in the utilisation of intermediate ergot alkaloid producing strains in pastures.

## 3. Materials and Methods

### 3.1. Bacterial Strains

*Escherichia coli* strain Top10 (Invitrogen Corp., Carlsbad, CA, USA) was grown on Luria–Bertani broths and agar plates supplemented with ampicillin (100 µg/mL).

### 3.2. Fungal Strains and Growth Conditions

Cultures of *E. festucae* var. *lolii* AR5 were maintained on 1.5% (*w*/*v*) potato dextrose agar (PDA) (Difco, Sparks, MD, USA) supplemented where necessary with hygromycin (150 µg/mL).

### 3.3. Plant Growth and Endophyte Inoculation

Endophyte-free seedlings of perennial ryegrass (*Lolium perenne* cv. Samson) were inoculated with *E. festucae* var. *lolii* AR5 wild type, ergot alkaloid deletion mutants, and ectopic controls using the method of Latch and Christensen [36]. Twenty plants representing five genotypes with four replicates were used per treatment. Seedlings were grown in proprietary potting mixture in 90 cm pots under glasshouse conditions for 6 weeks and assessed for endophyte infection by immunoblotting [37].

### 3.4. Genomic DNA and Plasmid Isolation

Genomic DNA was isolated from freeze-dried *Epichloë* mycelium as previously described [38]. Plasmid DNA was isolated and purified using a plasmid mini kit (Invitrogen).

### 3.5. Preparation of Gene Deletion Constructs

Gene deletion constructs for *dmaW*, *easG*, *cloA*, and *lpsB* were created by means of MultiSite Gateway cloning (Invitrogen) and utilized a split marker system [39] for improved homologous recombination frequencies. Flanking sequences for each gene were amplified by PCR with attB1- and attB2-tailed primers (Supplementary Figure S1 and Supplementary Table S1 from *E. Festucae* var. *lolii* AR5 genomic DNA. These fragments were recombined into pDONR SML and pDONR SMR vectors using BP clonase (invitrogen). Linear PCR products used for transformation were amplified using PrimeSTAR polymerase (Takara, Shiga, Japan) and primers listed in Supplementary Table S1.

### 3.6. Fungal Protoplasting and Transformation

Protoplasts of *E. festucae* var. *lolii* AR5 were prepared as described in Fleetwood et al., 2007 [22]. Fungal cultures were grown in 50 mL defined media [40] inoculated with macerated fungi (grown on cellophane PDA plates for 7 days at 22 °C) and grown at 22 °C with moderate shaking (150 rpm) for 5 days. The mycelial pellets were washed with 1 L sterile H_2_0 followed by a wash in OM buffer (1.2 M MgSO_4_, 10 mM Na_2_HPO_4_, pH 5.8). The washed mycelia were mixed with 30 mL of sterile OM buffer containing 15 mg/mL trichoderma lysing enzyme (Sigma, St Louis, MO, USA). This mixture was shaken gently (100 rpm) for 18 h at 30 °C. The digested hyphae were filtered through Mira-cloth (Calbiochem, San Diego, CA, USA) and the filtrate, containing the protoplasts, was overlaid with 2 mL STC buffer (0.6 M sorbitol, 100 mM Tris-HCL pH 8.0). The protoplasts were banded at the interface by centrifugation at 3000× *g* for 15 min, washed three times with 10 mL STC buffer (1 M sorbitol, 50 mM CaCl_2_, 50 mM Tris/HCl pH8.0) by centrifuging at 7700× *g* and resuspended in STC buffer to a final concentration of 1.25 × 10^8^ per mL. Protoplasts were transformed as described in Fleetwood et al., 2007 [22] with 100 fmol linear DNA PCR products for each of the gene deletion constructs. Transformants were selected on RG Media (PD with 0.8 M sucrose pH 6.5) containing hygromycin (150 µg/mL). To obtain clonal isolates, the resulting transformants were purified by sub-culturing three times as described by Young et al., 2005 [41].

### 3.7. Molecular Analysis of Transformants

Transformants were screened by PCR for homologous recombination using primers (Supplementary Table S1) annealed to sequences in the replacement construct on either side of the hygromycin cassette (Supplementary Figure S1 and Table S1).

### 3.8. Black Beetle Insect Feeding Assay

Plants, confirmed as endophyte-infected by immunoblotting along with uninfected controls, were placed in four replicate blocks in a glasshouse in March (autumn) 2017. Within each block, plants were placed in a row-column design with all treatments to a plant genotype randomized within a row and each column represented by a different plant genotype. Dead tillers and leaf sheath material were removed and plants were trimmed to 50 mm height and watered with 70 mL of water. On the same day, one male and one female black beetle that had been collected from pitfall traps set in a Waikato dairy pasture over the previous two weeks were caged onto each individual plant in the first two replicates using a fine nylon net sealed over a wire frame. The following day, beetles were caged onto the remaining two replicates. After two weeks, the cages were removed and herbage was cut from the plants just below ground level. The number of live and dead tillers that were damaged or undamaged by black beetle was recorded. Damaged tillers were scored on a scale of 1–3, where 1 = minor surface feeding on the outside of the tiller, 2 = moderate feeding that had partially penetrated the tiller, and 3 = severe feeding where the base of the tiller had been shredded and the tiller had wilted. The bottom 40 mm of tillers from each plant was removed and frozen in liquid nitrogen, and then subsequently freeze-dried for chemical analysis.

Throughout the experiment, plants were each watered with up to 140 mL, as needed.

### 3.9. Sample Preparation for Chemical Analysis

Basal sections of tillers were harvested into liquid nitrogen and then transferred to a freeze-drier. Lyophilized samples were ground and homogenized with a bead ruptor (Omni Bead Ruptor 24, Omni International Inc., Kennesaw, GA, USA) in a 7 mL vial using a ¼ inch zirconium bead (30 s at 4.5 m/s).

For analysis, sub-samples (50 mg) were extracted with 1 mL of extraction solvent (80% v/v methanol with 0.54 ng/mL ergotamine, 0.202 ng/mL festuclavine, and 1.7 ng/mL homoperamine as internal standard) in 2 mL plastic vials for 1 h by end-over-end rotation (30 Hz) in the dark. After centrifuging (5000× *g*, 5 min), 600 µL of the supernatant was further diluted with 3.2 mL of Milli-Q water (Millipore Australia Pty Ltd, North Ryde, NSW 2113, Australia) before undergoing solid-phase extraction clean-up, as described in Section 3.10. Standards were treated in a similar manner.

### 3.10. Solid-Phase Extraction Clean-Up

The diluted sample extracts were loaded onto pre-conditioned and pre-equilibrated (methanol and water respectively) Strata-X-CW SPE cartridges (60 mg/3 mL tubes, Phenomenex, Torrance, CA, USA) by centrifuging at 500× *g* for 2 min. The cartridges were washed with water (2 mL) and 50% methanol (1 mL) before the target analytes were collected by eluting with 750 µL of 5% formic acid in methanol and 750 µL of 1% ammonia in methanol. The eluents were collected in a high performance liquid chromatography (HPLC) vial and dried by centrifugal evaporation (Speed-Vac Plus SC110A, Savant Instruments Inc., Farmingdale, NY, USA) before resuspending in 200 µL of 50% methanol. Resuspended extracts were stored at −20 °C prior to analysis.

### 3.11. Analysis for Epichloë Ergot Alkaloids

Samples (10 µL injection) were chromatographically separated on a Kinetic C18 150 × 2.1 mm (2.6 µm) column (Phenomenex, Torrance, CA, USA) using the following linear gradient profile (eluent A, aqueous 0.1% formic acid and eluent B, acetonitrile with 0.1% formic acid); time 0 min (T_0_) at 10% B, T_6_ at 60% B, T_17_ at 100% B, and T_19_ at 100% B, followed by equilibration to initial conditions over the following 8 min. Detection and quantitation were achieved using a triple-quadrupole mass spectrometer (TSQ Quantum, Thermo Fisher Scientific, Waltham, MA, USA) with the following parameters: heated electrospray ionization (HESI) in positive ion mode, capillary temperature 270 °C, sheath gas pressure 60, ion sweep gas pressure 10, aux gas pressure 35, and a 0.7 amu window for both Q1 and Q3. Supplementary Table S4 shows the parameters specific to each compound.

### 3.12. Data Processing

The raw data were processed using LCQuan 2.7 (Thermo Fisher Scientific, Waltham, MA, USA). Where internal standards were used, LCQuan was used to determine the final concentration in the samples. 

### 3.13. Methods of Statistical Analysis

Statistical analysis was performed with Genstat, 19th edition. The proportion of all tillers showing black beetle damage scores greater than 1 was compared using a regression analysis of binomial data.

## Figures and Tables

**Table 1:** Mass spectrometry analysis of ergot and peramine alkaloids from perennial ryegrass infected with gene deletion strains. WT = wild type, E Free = uninfected perennial ryegrass. Lysergic acid is relative to ergovaline.

	Chanoclavine (mg/kg)	Agroclavine (mg/kg)	Elymoclavine (mg/kg)	Lysergic Acid (Relative to Ev)	Ergovaline (mg/kg)	Peramine (mg/kg)
AR5 *dmaW* KO15	0.00	0.00	0.00	0.00	0.00	29.76
AR5 *dmaW* KO20	0.00	0.00	0.00	0.00	0.00	22.60
AR5 *easG* KO3	0.33	0.00	0.00	0.00	0.00	16.18
AR5 *easG* KO20	0.69	0.00	0.00	0.00	0.00	21.38
AR5 *cloA* KO6	0.85	0.14	0.00	0.00	0.00	22.10
AR5 *cloA* KO32	0.86	0.18	0.00	0.00	0.00	25.71
AR5 *lpsB* KO10	0.89	0.02	0.00	0.71	0.00	25.78
AR5 *lpsB* KO11	0.85	0.02	0.00	0.49	0.00	31.62
AR5 *dmaW* E1	0.77	0.03	0.00	0.00	17.42	31.59
AR5 *easG* E14	0.88	0.03	0.00	0.30	22.10	28.81
AR5 *cloA* E4	0.87	0.04	0.00	0.00	13.42	27.64
AR5 *lpsB* E9	1.16	0.02	0.00	0.14	14.01	25.47
AR5 WT	0.97	0.01	0.00	0.05	4.50	9.24
E free	0.00	0.00	0.00	0.00	0.00	0.00

## Data Availability

Not applicable. Data is provided in the manuscript.

## Supplementary Materials

The following are available online at https://www.mdpi.com/2072-6651/13/2/76/s1, Figure S1: Gene deletion by homologous recombination and PCR screening strategy; Table S1: PCR amplicon sizes used to screen transformants for homologous recombination or ectopic insertion events; Table S2: Mass spectrometry raw data; Table S3: *E. festucae* M3 and *E. festucae* var. *lolii* AR5 gene models with AR5 NCBI accession numbers; Table S4: Mass spectrometer parameters for individual compounds.
