# Supplementary material for: Genetic Manipulation of the Ergot Alkaloid Pathway in Epichloë festucae var. lolii and Its Effect on Black Beetle Feeding Deterrence
Source: Toxins (Basel). 2021 Jan 20;13(2):76. doi: 10.3390/toxins13020076 (PMC7909537; doi:10.3390/toxins13020076)
Supplement: Supplementary file 1 [file toxins-13-00076-s001.pdf]

Supplementary Materials: Genetic Manipulation of the Ergot Alkaloid Pathway in *Epichloë festucae* var. *lolii* and Its Effect on Black Beetle Feeding Deterrence

Debbie Hudson, Wade Mace, Alison Popay, Joanne Jensen, Catherine McKenzie, Catherine Cameron and Richard Johnson

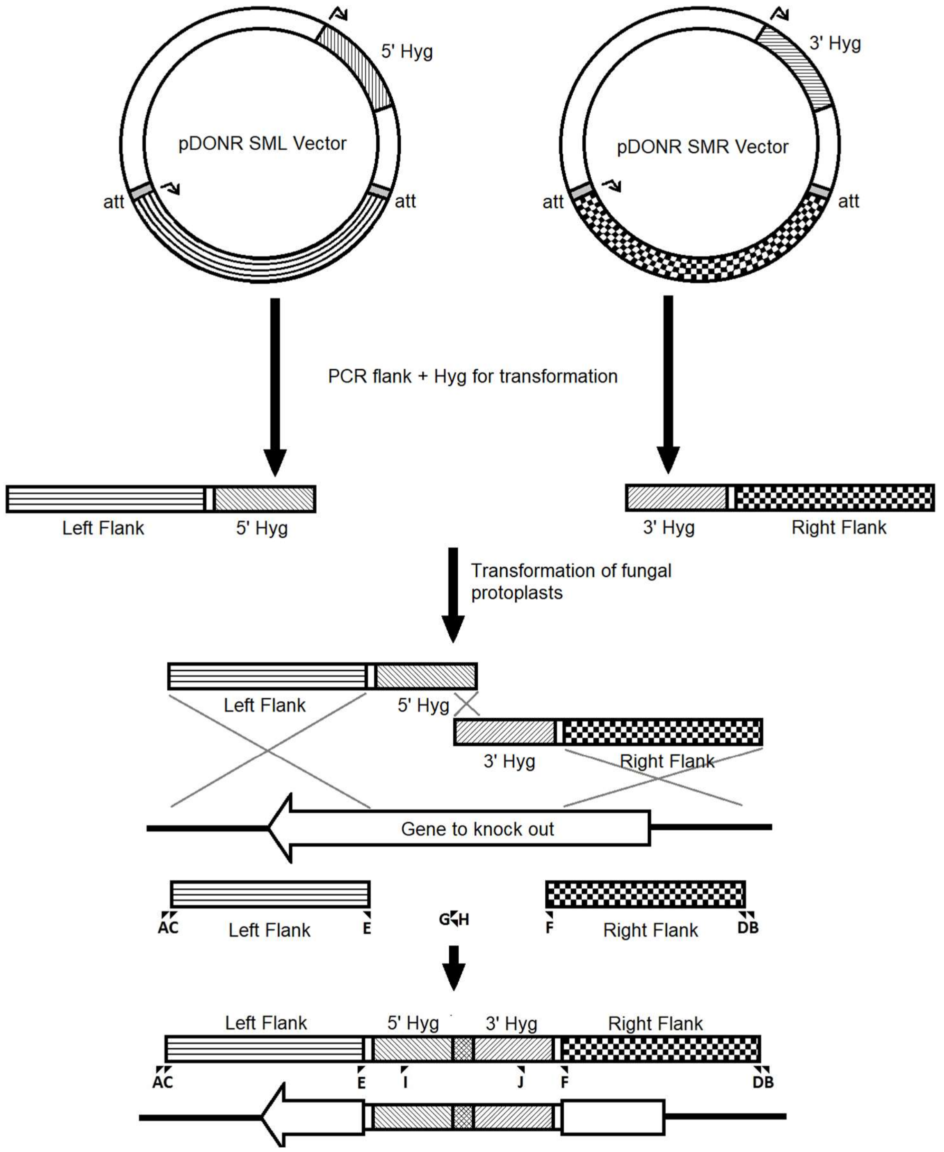

Figure S1. Gene deletion by homologous recombination and PCR screening strategy. Left and right flanking sequences of the gene to be deleted were amplified by PCR with attB1- and attB2-tailed primers (Supplementary Table S1) and cloned into pDONR SML and pDONR SMR split marker vectors [1] prior to transformation. Hygromycin resistant transformants were screened for homologous recombination or ectopic insertion events using primers A–J as illustrated (Supplementary Table S1). att = site-specific attachment sites; hyg = hygromycin resistance gene.

Table S1. PCR amplicon sizes used to screen transformants for homologous recombination or ectopic insertion events.

dmaW **Primer Name**	**Primer Sequence**	**Product Size**
dmaW **Left Flank F**	GGGGACCACTTTGTACAAGAAAGCTGGGTACTAAATACACGCCATACCTAACGC	1217bp
dmaW Left Flank R	GGGGACAAGTTTGTACAAAAAAGCAGGCTTAGCTTGTGTTTGGCTCCGTC	
dmaW Right Flank F	GGGGACAAGTTTGTACAAAAAAGCAGGCTTACCTGGTGGAGTGTCTTTGCC	1780bp
dmaW Right Flank R	GGGGACCACTTTGTACAAGAAAGCTGGGTAGCTAAGCCATTTCTCCACCTAACA	
dmaW SML KO PCR	CTAAATACACGCCATACCTAACGC	2756bp
SML Hyg KO PCR	GTCCATCACAGTTTGCCAGT	
dmaW SMR KO PCR	GCTAAGCCATTTCTCCACCTAACA	3587bp
SMR Hyg KO PCR	CGATGTAGGAGGGCGTGGAT	

dmaW **Primer Name**	**Primer Sequence**	**Product Size**		
		**WT**	**KO**	**Ectopic**
(A) *dmaW* 3F	AGCCTAAAGGTTCTCTAAAG	3918bp	6416bp	3918bp
(B) *dmaW* 3921R	GGATTAGACCTTATCTCTT			
(C) *dmaW* SML KO PCR	CTAAATACACGCCATACCTAACGC	3458bp	5956bp	both
(D) *dmaW* SMR KO PCR	GCTAAGCCATTTCTCCACCTAACA			
(E) *dmaW* 1252F	GACGGAGCCAAACACAAGC	622bp	3120bp	both
(F) *dmaW* 1854R	GGCAAAGACACTCCACCAGG			
(E) *dmaW* 1252F	GACGGAGCCAAACACAAGC	318bp	-	318bp
(G) *dmaW* 1570R	CCTATTAACGCCGCAACTGG			
(F) *dmaW* 1854R	GGCAAAGACACTCCACCAGG	324bp	-	324bp
(H) *dmaW* 1550F	CCAGTTGCGGCGTTAATAGG			
(F) *dmaW* 1854R	GGCAAAGACACTCCACCAGG	-	2050bp	2050bp
(I) MG118	TTCCCACTTCATCGCAGCTTG			
(E) *dmaW* 1252F	GACGGAGCCAAACACAAGC	-	2084bp	2084bp
(J) MG119	TACACAGCCATCGGTCCAGAC			
(I) MG118	TTCCCACTTCATCGCAGCTTG	-	1014bp	1014bp
(J) MG119	TACACAGCCATCGGTCCAGAC			

easC **Primer Name**	**Primer Sequence**	**Product Size**

easG Left Flank F	GGGGACCACTTTGTACAAGAAAGCTGGGTACACAGCATCAACTACCTCTTTCCA	2031bp		
easG Left Flank R	GGGGACAAGTTTGTACAAAAAAGCAGGCTTAAGATGGGTGGCGGAACAAG			
easG Right Flank F	GGGGACAAGTTTGTACAAAAAAGCAGGCTTAAGATATTGCCCGTGTCGCAT	1888bp		
easG Right Flank R	GGGGACCACTTTGTACAAGAAAGCTGGGTATGCGGAAAAGACAACGGGT			
easG SML KO PCR	CACAGCATCAACTACCTCTTTCCA	3570bp		
SML Hyg KO PCR	GTCCATCACAGTTTGCCAGT			
easG SMR KO PCR	TGCGGAAAAGACAACGGGT	3695bp		
SMR Hyg KO PCR	CGATGTAGGAGGGCGTGGAT			
***easC* Primer Name**	**Primer Sequence**	**Product Size**		
		**WT**	**KO**	**Ectopic**
(A) *easG* 3429F	GCTTGAACGAAATCACCGATG	4374bp	7092bp	4374bp
(B) *easG* 7866R	CCGCCTAGTATCTGCCACATTAC			
(C) *easG* SML KO PCR	CACAGCATCAACTACCTCTTTCCA	4160bp	6878bp	both
(D) *easG* SMR KO PCR	TGCGGAAAAGACAACGGGT			
(E) *easG* 5631F	CTTGTTCCGCCACCCATCT	402bp	3120bp	both
(F) *easG* 6013R	ATGCGACACGGGCAATATCT			
(E) *easG* 5631F	CTTGTTCCGCCACCCATCT	143bp	-	143bp
(G) *easG* 5774R	TCGTGAACCTGTCCCATG			
(F) *easG* 6013R	ATGCGACACGGGCAATATCT	277bp	-	277bp
(H) *easG* 5756F	CATGGGACAGGTTCACGA			
(F) *easG* 6013R	ATGCGACACGGGCAATATCT	-	2050bp	2050bp
(I) MG118	TTCCCACTTCATCGCAGCTTG			
(E) *easG* 5631F	CTTGTTCCGCCACCCATCT	-	2084bp	2084bp
(J) MG119	TACACAGCCATCGGTCCAGAC			
(I) MG118	TTCCCACTTCATCGCAGCTTG	-	1014bp	1014bp
(J) MG119	TACACAGCCATCGGTCCAGAC			
***cloA* Primer Name**	**Primer Sequence**	**Product Size**		
cloA Left Flank F	GGGGACCACTTTGTACAAGAAAGCTGGGTAGATCCTAGTTTAACTTGCTTCCCG	1160bp		
cloA Left Flank R	GGGGACAAGTTTGTACAAAAAAGCAGGCTTATGGAACGACGAGGATGAACG			
cloA Right Flank F	GGGGACAAGTTTGTACAAAAAAGCAGGCTTAATCCTTTCGGCAGTTACCACG	1206bp		
cloA Right Flank R	GGGGGACCACTTTGTACAAGAAAGCTGGGTATCCGTTCTTCTTGCGTGCC			

cloA SML KO PCR	GATCCTAGTTTAACTTGCTTCCCG	2699bp
SML Hyg KO PCR	GTCCATCACAGTTTGCCAGT	
cloA SMR KO PCR	TCCGTTCTTCTTGCGTGCC	3013bp
SMR Hyg KO PCR	CGATGTAGGAGGGCGTGGAT	

***cloA* Primer Name**	**Primer Sequence**	**Product Size**		
		**WT**	**KO**	**Ectopic**
(A) *cloA* 467F	CCTCACACTTCATAGGGTTAGC	2790bp	5391bp	2790bp
(B) *cloA* 3259R	GTGAGGATATTACGGCATA			
(C) *cloA* SML KO PCR	GATCCTAGTTTAACTTGCTTCCCG	2724bp	5325bp	both
(D) *cloA* SMR KO PCR	TCCGTTCTTCTTGCGTGCC			
(E) *cloA* 1590F	CGTTCATCCTCGTCGTTCCA	521bp	3122bp	both
(F) *cloA* 2090R	CGTGGTAACTGCCGAAAGGAT			
(E) *cloA* 1590F	CGTTCATCCTCGTCGTTCCA	387bp	-	387bp
(G) *cloA* 1977R	GGGAATAGACCTCAATGATGC			
(F) *cloA* 2090R	CGTGGTAACTGCCGAAAGGAT	155bp	-	155bp
(H) *cloA* 1956F	GCATCATTGAGGTCTATTCCC			
(F) *cloA* 2090R	CGTGGTAACTGCCGAAAGGAT	-	2051bp	2051bp
(I) MG118	TTCCCACTTCATCGCAGCTTG			
(E) *cloA* 1590F	CGTTCATCCTCGTCGTTCCA	-	2085bp	2085bp
(J) MG119	TACACAGCCATCGGTCCAGAC			
(I) MG118	TTCCCACTTCATCGCAGCTTG	-	1014bp	1014bp
(J) MG119	TACACAGCCATCGGTCCAGAC			

***lpsB* Primer Name**	**Primer Sequence**	**Product Size**
lpsB Left Flank F	GGGGACCACTTTGTACAAGAAAGCTGGGTAGTAGGGGCGACATTAAGAA	1658bp
lpsB Left Flank R	GGGGACAAGTTTGTACAAAAAAGCAGGCTTAGCCGCTTGGATTCTCTGGA	
lpsB Right Flank F	GGGGACAAGTTTGTACAAAAAAGCAGGCTTACCCCGATTCTTGTGGCTTTC	1996bp
lpsB Right Flank R	GGGGACCACTTTGTACAAGAAAGCTGGGTACTCACCGTCATGTCGCAG	
lpsB SML KO PCR	GTAGGGGCGACATTAAGAAGGC	3197bp
SML Hyg KO PCR	GTCCATCACAGTTTGCCAGT	
lpsB SMR KO PCR	CTCACCGTCATGTCGCAG	3803bp
SMR Hyg KO PCR	CGATGTAGGAGGGCGTGGAT	

***lpsB* Primer Name**	**Primer Sequence**	**Product Size**

		WT	KO	Ectopic
(A) *lpsB* 10050F<br>(B) *lpsB* 15631R	TATGCTCATTGCGACGGAG<br>CCCTTGATTTCGCTTGTTGC	5581bp	6486bp	5581bp
(C) *lpsB* SML KO PCR<br>(D) *lpsB* SMR KO PCR	GTAGGGGCGACATTAAGAAGGC<br>CTCACCGTCATGTCGCAG	5348bp	6613bp	both
(E) *lpsB* 11649F<br>(F) *lpsB* 13504R	TCCAGAGAATCCAAGCGGC<br>GAAAGCCACAAGAATCGGGG	1855bp	3120bp	both
(E) *lpsB* 11649F<br>(G) *lpsB* 12353R	TCCAGAGAATCCAAGCGGC<br>CAAGCATAGCGACAGTGGTC	704bp	-	704bp
(F) *lpsB* 13504R<br>(H) *lpsB* 12333F	GAAAGCCACAAGAATCGGGG<br>GACCACTGTCGCTATGCTTG	1171bp	-	1171bp
(F) *lpsB* 13504R<br>(I) MG118	GAAAGCCACAAGAATCGGGG<br>TTCCCACTTCATCGCAGCTTG	-	2050bp	2050bp
(E) *lpsB* 11649F<br>(J) MG119	TCCAGAGAATCCAAGCGGC<br>TACACAGCCATCGGTCCAGAC	-	2084bp	2084bp
(I) MG118<br>(J) MG119	TTCCCACTTCATCGCAGCTTG<br>TACACAGCCATCGGTCCAGAC	-	1014bp	1014bp

The letters A-J refer to primers used to screen transformants as illustrated in Supplementary Figure 1.

Table S2. Mass spectrometry raw data.

Fungal strain	Plant Number	Chanoclavine (mg/kg)	Agroclavine (mg/kg)	Elymoclavine (mg/kg)	Lysergic Acid (relative to Ev)	Ergovaline (mg/kg)	Peramine (mg/kg)
AR5 *dmaW* KO15	R3	0.00	0.00	0.00	0.00	0.00	41.6
AR5 *dmaW* KO15	R13	0.00	0.00	0.00	0.00	0.00	32.9
AR5 *dmaW* KO15	R30	0.00	0.00	0.00	0.00	0.00	29.8
AR5 *dmaW* KO15	R43	0.00	0.00	0.00	0.00	0.00	16.3
AR5 *dmaW* KO15	R51	0.00	0.00	0.00	0.00	0.00	18.2
AR5 *dmaW* KO15	R60	0.00	0.00	0.00	0.00	0.00	27.3
AR5 *dmaW* KO15	R69	0.00	0.00	0.00	0.00	0.00	17.8
AR5 *dmaW* KO15	R83	0.00	0.00	0.00	0.00	0.00	28.7
AR5 *dmaW* KO15	R99	0.00	0.00	0.00	0.00	0.00	16.6
AR5 *dmaW* KO15	R104	0.00	0.00	0.00	0.00	0.00	54.8
AR5 *dmaW* KO15	R117	0.00	0.00	0.00	0.00	0.00	30.7
AR5 *dmaW* KO15	R122	0.00	0.00	0.00	0.00	0.00	29.4
AR5 *dmaW* KO15	R138	0.00	0.00	0.00	0.00	0.00	26.2
AR5 *dmaW* KO15	R152	0.00	0.00	0.00	0.00	0.00	26.7
AR5 *dmaW* KO15	R164	0.00	0.00	0.00	0.00	0.00	70.5
AR5 *dmaW* KO15	R174	0.00	0.00	0.00	0.00	0.00	41.0
AR5 *dmaW* KO15	R178	0.00	0.00	0.00	0.00	0.00	12.2
AR5 *dmaW* KO15	R195	0.00	0.00	0.00	0.00	0.00	25.1
AR5 *dmaW* KO15	R209	0.00	0.00	0.00	0.00	0.00	28.1
AR5 *dmaW* KO15	R213	0.00	0.00	0.00	0.00	0.00	21.2
AR5 *dmaW* KO20	R8	0.00	0.00	0.00	0.00	0.00	34.1
AR5 *dmaW* KO20	R21	0.00	0.00	0.00	0.00	0.00	37.4
AR5 *dmaW* KO20	R24	0.00	0.00	0.00	0.00	0.00	18.0
AR5 *dmaW* KO20	R52	0.00	0.00	0.00	0.00	0.00	0.0
AR5 *dmaW* KO20	R66	0.00	0.01	0.00	0.00	0.00	14.7
AR5 *dmaW* KO20	R67	0.00	0.00	0.00	0.00	0.00	30.6
AR5 *dmaW* KO20	R84	0.00	0.00	0.00	0.00	0.00	25.6
AR5 *dmaW* KO20	R91	0.00	0.00	0.00	0.00	0.00	13.5
AR5 *dmaW* KO20	R103	0.00	0.00	0.00	0.00	0.00	7.2

AR5 *dmaW* KO20	R121	0.00	0.00	0.00	0.00	0.00	28.3
AR5 *dmaW* KO20	R130	0.00	0.00	0.00	0.00	0.00	12.8
AR5 *dmaW* KO20	R136	0.00	0.00	0.00	0.00	0.00	22.5
AR5 *dmaW* KO20	R144	0.00	0.00	0.00	0.00	0.00	15.7
AR5 *dmaW* KO20	R156	0.00	0.00	0.00	0.00	0.00	35.3
AR5 *dmaW* KO20	R170	0.00	0.00	0.00	0.00	0.00	37.4
AR5 *dmaW* KO20	R186	0.00	0.00	0.00	0.00	0.00	21.4
AR5 *dmaW* KO20	R194	0.00	0.00	0.00	0.00	0.00	28.9
AR5 *dmaW* KO20	R201	0.00	0.00	0.00	0.00	0.00	18.5
AR5 *dmaW* KO20	R215	0.00	0.00	0.00	0.00	0.00	27.4
AR5 *easG* KO3	R11	0.36	0.01	0.00	0.00	0.00	28.2
AR5 *easG* KO3	R19	0.42	0.00	0.00	0.00	0.00	27.9
AR5 *easG* KO3	R25	0.13	0.00	0.00	0.00	0.00	17.4
AR5 *easG* KO3	R34	1.12	0.00	0.00	0.00	0.00	14.4
AR5 *easG* KO3	R50	0.38	0.00	0.00	0.00	0.00	9.2
AR5 *easG* KO3	R62	0.30	0.00	0.00	0.00	0.00	11.1
AR5 *easG* KO3	R70	0.00	0.00	0.00	0.00	0.00	14.9
AR5 *easG* KO3	R79	0.17	0.00	0.00	0.00	0.00	13.6
AR5 *easG* KO3	R98	0.30	0.00	0.00	0.00	0.00	40.8
AR5 *easG* KO3	R105	0.13	0.00	0.00	0.00	0.00	5.1
AR5 *easG* KO3	R120	0.53	0.00	0.00	0.00	0.00	25.9
AR5 *easG* KO3	R129	0.27	0.00	0.00	0.00	0.00	19.7
AR5 *easG* KO3	R137	0.06	0.00	0.00	0.00	0.00	7.3
AR5 *easG* KO3	R148	0.30	0.01	0.00	0.00	0.00	12.1
AR5 *easG* KO3	R155	0.11	0.00	0.00	0.00	0.00	8.6
AR5 *easG* KO3	R168	0.57	0.00	0.00	0.00	0.00	12.9
AR5 *easG* KO3	R187	0.36	0.00	0.00	0.00	0.00	5.6
AR5 *easG* KO3	R196	0.22	0.00	0.00	0.00	0.00	11.1
AR5 *easG* KO3	R200	0.29	0.00	0.00	0.00	0.00	28.0
AR5 *easG* KO3	R218	0.67	0.00	0.00	0.00	0.00	9.8
AR5 *easG* KO20	R9	0.39	0.00	0.00	0.00	0.00	26.1
AR5 *easG* KO20	R18	1.47	0.00	0.00	0.00	0.00	54.3
AR5 *easG* KO20	R45	1.23	0.00	0.00	0.00	0.00	19.1

AR5 *easG* KO20	R61	0.29	0.00	0.00	0.00	0.00	5.1
AR5 *easG* KO20	R74	0.37	0.00	0.00	0.00	0.00	18.0
AR5 *easG* KO20	R87	0.35	0.00	0.00	0.00	0.00	12.8
AR5 *easG* KO20	R92	0.83	0.00	0.00	0.00	0.00	18.4
AR5 *easG* KO20	R110	0.84	0.00	0.00	0.00	0.00	15.4
AR5 *easG* KO20	R115	0.61	0.01	0.00	0.00	0.00	5.0
AR5 *easG* KO20	R124	0.84	0.01	0.00	0.00	0.00	46.5
AR5 *easG* KO20	R139	0.31	0.01	0.00	0.00	0.00	17.1
AR5 *easG* KO20	R153	0.91	0.00	0.00	0.00	0.00	30.4
AR5 *easG* KO20	R158	0.60	0.00	0.00	0.00	0.00	22.1
AR5 *easG* KO20	R173	0.78	0.00	0.00	0.00	0.00	12.8
AR5 *easG* KO20	R179	0.97	0.00	0.00	0.00	0.00	21.2
AR5 *easG* KO20	R188	0.29	0.00	0.00	0.00	0.00	10.6
AR5 *easG* KO20	R204	0.45	0.00	0.00	0.00	0.00	36.4
AR5 *easG* KO20	R211	0.83	0.01	0.00	0.00	0.00	13.4
AR5 *cloA* KO6	R5	0.90	0.83	0.00	0.00	0.00	26.9
AR5 *cloA* KO6	R17	1.45	0.16	0.00	0.00	0.00	24.6
AR5 *cloA* KO6	R26	0.85	0.12	0.00	0.00	0.00	23.3
AR5 *cloA* KO6	R40	0.94	0.10	0.00	0.00	0.00	16.5
AR5 *cloA* KO6	R53	0.59	0.07	0.00	0.00	0.00	27.6
AR5 *cloA* KO6	R64	0.38	0.06	0.00	0.00	0.00	13.4
AR5 *cloA* KO6	R68	0.63	0.13	0.00	0.00	0.00	15.0
AR5 *cloA* KO6	R80	0.70	0.14	0.00	0.00	0.00	32.1
AR5 *cloA* KO6	R96	0.78	0.06	0.00	0.00	0.00	22.6
AR5 *cloA* KO6	R100	0.62	0.11	0.00	0.00	0.00	14.1
AR5 *cloA* KO6	R111	1.61	0.26	0.00	0.00	0.00	24.8
AR5 *cloA* KO6	R125	1.38	0.06	0.00	0.00	0.00	28.4
AR5 *cloA* KO6	R142	0.85	0.14	0.00	0.00	0.00	20.6
AR5 *cloA* KO6	R149	0.37	0.03	0.00	0.00	0.00	13.7
AR5 *cloA* KO6	R165	0.71	0.16	0.00	0.00	0.00	22.5
AR5 *cloA* KO6	R167	0.75	0.09	0.00	0.00	0.00	36.7
AR5 *cloA* KO6	R181	0.83	0.11	0.00	0.00	0.00	27.3
AR5 *cloA* KO6	R198	0.73	0.04	0.00	0.00	0.00	16.4

AR5 *cloA* KO6	R208	0.95	0.03	0.00	0.00	0.00	22.6
AR5 *cloA* KO6	R216	0.90	0.05	0.00	0.00	0.00	13.0
AR5 *cloA* KO32	R10	1.04	0.21	0.00	0.00	0.00	27.8
AR5 *cloA* KO32	R15	0.85	0.25	0.00	0.00	0.00	17.0
AR5 *cloA* KO32	R31	1.42	0.31	0.00	0.00	0.00	28.1
AR5 *cloA* KO32	R44	0.56	0.19	0.00	0.00	0.00	15.6
AR5 *cloA* KO32	R46	0.74	0.18	0.00	0.00	0.00	32.5
AR5 *cloA* KO32	R63	0.38	0.11	0.00	0.00	0.00	26.5
AR5 *cloA* KO32	R73	0.52	0.22	0.00	0.00	0.00	29.9
AR5 *cloA* KO32	R86	1.06	0.26	0.00	0.00	0.00	26.1
AR5 *cloA* KO32	R89	0.78	0.10	0.00	0.00	0.00	22.5
AR5 *cloA* KO32	R101	0.71	0.09	0.00	0.00	0.00	16.0
AR5 *cloA* KO32	R116	1.21	0.45	0.00	0.00	0.00	60.4
AR5 *cloA* KO32	R128	0.38	0.10	0.00	0.00	0.00	13.6
AR5 *cloA* KO32	R135	0.31	0.08	0.00	0.00	0.00	17.2
AR5 *cloA* KO32	R154	1.08	0.14	0.00	0.00	0.00	28.9
AR5 *cloA* KO32	R162	1.06	0.20	0.00	0.00	0.00	29.3
AR5 *cloA* KO32	R171	1.04	0.33	0.00	0.00	0.00	29.9
AR5 *cloA* KO32	R177	0.56	0.10	0.00	0.00	0.00	14.3
AR5 *cloA* KO32	R192	0.75	0.08	0.00	0.00	0.00	37.5
AR5 *cloA* KO32	R202	2.25	0.09	0.00	0.00	0.00	17.5
AR5 *cloA* KO32	R214	0.62	0.10	0.00	0.00	0.00	23.4
AR5 *lpsB* KO10	R2	0.78	0.03	0.00	0.64	0.00	23.5
AR5 *lpsB* KO10	R14	1.99	0.04	0.00	0.83	0.00	35.3
AR5 *lpsB* KO10	R23	0.63	0.03	0.00	0.56	0.00	40.4
AR5 *lpsB* KO10	R38	0.54	0.01	0.00	0.83	0.00	24.1
AR5 *lpsB* KO10	R54	0.74	0.00	0.00	0.51	0.00	21.7
AR5 *lpsB* KO10	R58	0.51	0.00	0.00	1.63	0.00	27.9
AR5 *lpsB* KO10	R76	0.54	0.00	0.00	1.43	0.00	15.1
AR5 *lpsB* KO10	R85	0.66	0.02	0.00	0.76	0.00	16.6
AR5 *lpsB* KO10	R93	0.50	0.02	0.00	1.14	0.00	24.1
AR5 *lpsB* KO10	R108	2.71	0.01	0.00	0.31	0.00	30.8
AR5 *lpsB* KO10	R112	0.72	0.06	0.00	1.30	0.00	38.6

AR5 *lpsB* KO10	R127	0.29	0.03	0.00	0.42	0.00	18.3
AR5 *lpsB* KO10	R143	0.30	0.01	0.00	0.05	0.00	13.6
AR5 *lpsB* KO10	R147	2.14	0.03	0.00	0.31	0.00	27.7
AR5 *lpsB* KO10	R161	0.62	0.03	0.00	0.38	0.00	41.5
AR5 *lpsB* KO10	R169	0.72	0.03	0.00	1.55	0.00	29.8
AR5 *lpsB* KO10	R184	0.61	0.01	0.00	0.36	0.00	17.6
AR5 *lpsB* KO10	R193	0.74	0.00	0.00	0.15	0.00	13.3
AR5 *lpsB* KO10	R207	1.52	0.02	0.00	0.43	0.00	29.3
AR5 *lpsB* KO10	R210	0.55	0.03	0.00	0.69	0.00	26.7
AR5 *lpsB* KO11	R6	0.46	0.02	0.00	0.73	0.00	45.2
AR5 *lpsB* KO11	R16	0.93	0.05	0.00	0.90	0.00	58.0
AR5 *lpsB* KO11	R42	0.68	0.01	0.00	0.22	0.00	29.4
AR5 *lpsB* KO11	R48	1.21	0.03	0.00	0.58	0.00	30.3
AR5 *lpsB* KO11	R65	0.88	0.04	0.00	0.55	0.00	27.5
AR5 *lpsB* KO11	R75	0.43	0.02	0.00	0.20	0.00	22.1
AR5 *lpsB* KO11	R78	0.59	0.00	0.00	0.19	0.00	11.4
AR5 *lpsB* KO11	R95	0.75	0.00	0.00	0.62	0.00	31.6
AR5 *lpsB* KO11	R102	1.03	0.03	0.00	0.87	0.00	50.6
AR5 *lpsB* KO11	R114	1.05	0.06	0.00	0.69	0.00	31.5
AR5 *lpsB* KO11	R126	1.52	0.04	0.00	0.45	0.00	59.2
AR5 *lpsB* KO11	R140	0.37	0.01	0.00	0.25	0.00	25.2
AR5 *lpsB* KO11	R145	1.09	0.02	0.00	0.20	0.00	23.8
AR5 *lpsB* KO11	R157	0.42	0.01	0.00	0.24	0.00	25.3
AR5 *lpsB* KO11	R175	0.61	0.02	0.00	0.91	0.00	12.2
AR5 *lpsB* KO11	R183	0.52	0.01	0.00	0.28	0.00	27.8
AR5 *lpsB* KO11	R189	1.07	0.03	0.00	0.67	0.00	42.2
AR5 *lpsB* KO11	R206	1.91	0.02	0.00	0.43	0.00	27.5
AR5 *lpsB* KO11	R220	0.62	0.02	0.00	0.35	0.00	20.2
AR5 *dmaW* E1	R55	0.92	0.05	0.00	0.00	13.87	20.3
AR5 *dmaW* E1	R71	0.44	0.01	0.00	0.00	17.10	23.1
AR5 *dmaW* E1	R133	0.32	0.04	0.00	0.00	11.85	59.3
AR5 *dmaW* E1	R176	0.63	0.02	0.00	0.00	19.11	15.5
AR5 *dmaW* E1	R191	1.56	0.03	0.00	0.00	25.15	39.7

AR5 *easG* E14	R1	0.68	0.03	0.00	0.13	8.76	23.8
AR5 *easG* E14	R36	0.89	0.02	0.00	0.69	32.51	44.7
AR5 *easG* E14	R109	0.80	0.00	0.00	0.07	15.35	26.7
AR5 *easG* E14	R113	1.31	0.07	0.00	0.22	41.88	27.0
AR5 *easG* E14	R182	0.73	0.03	0.00	0.40	12.00	21.9
AR5 *cloA* E4	R20	0.60	0.07	0.00	0.00	11.53	11.7
AR5 *cloA* E4	R81	0.50	0.02	0.00	0.00	5.52	17.1
AR5 *cloA* E4	R123	1.70	0.05	0.00	0.00	34.26	74.8
AR5 *cloA* E4	R159	0.56	0.03	0.00	0.00	8.77	18.7
AR5 *cloA* E4	R205	0.99	0.01	0.00	0.00	7.04	15.8
AR5 *lpsB* E9	R29	1.57	0.02	0.00	0.01	19.63	26.4
AR5 *lpsB* E9	R57	0.69	0.06	0.00	0.42	13.00	24.2
AR5 *lpsB* E9	R97	0.69	0.00	0.00	0.04	8.75	28.3
AR5 *lpsB* E9	R151	1.16	0.03	0.00	0.17	12.04	32.7
AR5 *lpsB* E9	R219	1.67	0.02	0.00	0.04	16.61	15.8
AR5 WT	R4	1.36	0.02	0.00	0.05	6.24	19.6
AR5 WT	R22	1.61	0.01	0.00	0.00	7.75	9.7
AR5 WT	R28	0.26	0.00	0.00	0.01	0.32	0.0
AR5 WT	R41	0.64	0.01	0.00	0.02	1.75	6.4
AR5 WT	R49	1.23	0.00	0.00	0.02	3.12	10.5
AR5 WT	R56	0.59	0.00	0.00	0.43	3.73	12.5
AR5 WT	R77	0.71	0.00	0.00	0.18	5.33	6.6
AR5 WT	R82	0.68	0.01	0.00	0.02	2.87	10.1
AR5 WT	R94	0.70	0.00	0.00	0.01	2.65	8.4
AR5 WT	R106	2.00	0.00	0.00	0.02	8.35	8.3
AR5 WT	R119	0.55	0.01	0.00	0.02	1.56	4.8
AR5 WT	R131	0.78	0.01	0.00	0.02	6.14	12.3
AR5 WT	R134	0.91	0.03	0.00	0.00	7.94	12.1
AR5 WT	R146	0.47	0.00	0.00	0.00	2.93	7.8
AR5 WT	R163	1.36	0.01	0.00	0.00	7.32	11.1
AR5 WT	R172	0.67	0.01	0.00	0.00	2.24	10.2
AR5 WT	R180	1.31	0.01	0.00	0.08	4.51	9.8
AR5 WT	R190	0.48	0.00	0.00	0.09	1.43	4.1

AR5 WT	R199	1.25	0.00	0.00	0.00	4.88	7.9
AR5 WT	R217	1.89	0.01	0.00	0.05	8.96	12.4
E free	R7	0.00	0.00	0.00	0.00	0.00	0.0
E free	R32	0.00	0.00	0.00	0.00	0.00	0.0
E free	R37	0.00	0.00	0.00	0.00	0.00	0.0
E free	R59	0.00	0.00	0.00	0.00	0.00	0.0
E free	R72	0.00	0.00	0.00	0.00	0.00	0.0
E free	R88	0.00	0.00	0.00	0.00	0.00	0.0
E free	R90	0.00	0.00	0.00	0.00	0.00	0.0
E free	R107	0.00	0.00	0.00	0.00	0.00	0.0
E free	R118	0.00	0.00	0.00	0.00	0.00	0.0
E free	R132	0.00	0.00	0.00	0.00	0.00	0.0
E free	R141	0.00	0.00	0.00	0.00	0.00	0.0
E free	R150	0.00	0.00	0.00	0.00	0.00	0.0
E free	R160	0.00	0.00	0.00	0.00	0.00	0.0
E free	R166	0.00	0.00	0.00	0.00	0.00	0.0
E free	R185	0.00	0.00	0.00	0.00	0.00	0.0
E free	R197	0.00	0.00	0.00	0.00	0.00	0.0

Table S3. *E. festucae* M3 and *E. festucae* var. *lolii* AR5 gene models with AR5 NCBI accession numbers.

Gene	M3 Gene Model *	AR5 Gene Model	AR5 GenBank Accession Number
dmaW	EfM3.065770	AR5G0114884	MW353150
easG	EfM3.049650	AR5G0109173	MW353151
cloA	EfM3.065760	AR5G0100604	MW353152
lpsB	EfM3.049620	AR5G0109168	MW353153

* Schardl et al., 2013. Plant-symbiotic fungi as chemical engineers: multi-genome analysis of the Clavicipitaceae reveals dynamics of alkaloid loci. PLoS Genet 9(2):e1003323, doi:10.1371/journal.pgen.1003323.

Table S4. Mass spectrometer parameters for individual compounds.

Compounds	Retention Time	Parent Ion	MRM (Q *)	CE	MRM (v ‡)	CE	Tube Lens	Dwell Time	LOD (μg/g)	LOQ (μg/g)
Peramine	2.96	248	206	10	175	16	94	0.025	0.1	0.3
Homoperamine (IS)	3.41	262	245	14	203	21	91	0.025		
Chanoclavine	3.63	257	168	17	226	7	72	0.025	0.01	0.05
Agroclavine	4.44	239	208	16	183	18	75	0.025	0.01	0.05
Elymoclavine	3.39	255	224	12	180	37	80	0.025	0.01	0.05
Lysergic acid	3.49	269	223	24	254	20	96	0.025	0.03	0.1
Festuclavine (IS)	4.51	241	168	26	210	30	75	0.025		
Ergovaline	4.81/4.91 #	534	223	30	516#	14	104	0.025	0.01	0.05
Ergotamine (IS)	5.35/5.42 #	582	223	30	564#	14	108	0.025		

* quantitation MRM; ‡ validation MRM; # For the stereo-isomers of ergovaline and ergotamine (ergovalinine and ergotaminine), the validation MRM was used for quantitation as the quantitation ion is not observed. (IS) Internal standard used to quantify the preceding compound/s.

References

1. Rahnama, M.; Forester, N.; Ariyawansa, K.; Voisey, C.; Johnson, L.; Johnson, R.; Fleetwood, D. Efficient targeted mutagenesis in *Epichloë festucae* using a split marker system. *J. Microbiol. Methods* **2017**, *134*, 62–65.
